# AN INTERDISCIPLINARY PEDAGOGICAL APPROACH THAT HARNESSES COLLABORATIVE NETWORKS

**DOI:** 10.1093/geroni/igz038.547

**Published:** 2019-11-08

**Authors:** Tobi A Abramson, Martha Siegel

**Affiliations:** 1 New York City Department for the Aging, New York, New York, United States; 2 New York Institute of Technology, Old Westbury, New York, United States

## Abstract

Opportunities abound in healthcare and non-healthcare for innovative pedagogy, products, and services to address the growth of the aging population and ageism. ‘Aging-in-place’ homes and healthcare settings, often ill-prepared to meet aging needs, require redesign to incorporate designs that are responsive to an older or disabled person’s needs. This pedagogical approach used interprofessional/cross-disciplinary collaborative learning to incorporate a person-centered approach to developing evidence-based interior design solutions to transform homes, healthcare environments, and communities. This pedagogical collaboration built networks across disciplines, provided a unique learning environment by focusing on student learning through engagement of cross-discipline undergraduate and graduate students and external collaborators (older adults, healthcare systems). All students participated in the live project within and outside of the classroom utilizing print materials, didactic learning, experiential activities, research, technology, site visits, client interviewing, recording data, team meetings, and designing and presenting a solution to a panel of judges. Assessments indicated students’ appreciation of the project for their professional development and an enhanced understanding of teamwork strategies and cross-disciplinary classrooms for interprofessional learning. This pedagogical approach broke down academic silos and deserves more attention in academia to prepare students with the skills to work with an aging population. As the population ages, it is essential that college courses include approaches to decrease ageism and lead to the building of the workforce. Collaborative learning builds the foundation and networks that will help emerging professionals meet the needs of an aging population.

